# Supplementary material for: Actinic Keratoses (AK): An Exploratory Questionnaire-Based Study of Patients’ Illness Perceptions
Source: Curr Oncol. 2022 Jul 21;29(7):5150–63. doi: 10.3390/curroncol29070408 (PMC9323725; doi:10.3390/curroncol29070408)
Supplement: Supplementary file 1 [file curroncol-29-00408-s001.zip › curroncol-1806609-supplementary update.pdf]

Supplementary Tables

Supplementary Table S1a. Illness perception dimensions

Rating scale	Duration of the disease n (%)	Intensity of symptoms n (%)	Understanding of the disease n (%)	Concern about the disease n (%)
1	43 (21.29)	107 (52.71)	30 (14.56)	80 (38.83)
2	9 (4.46)	25 (12.32)	13 (6.31)	13 (6.31)
3	20 (9.90)	22 (10.84)	11 (5.34)	18 (8.74)
4	3 (1.49)	5 (2.46)	6 (2.91)	6 (2.91)
5	23 (11.39)	15 (7.39)	10 (4.85)	23 (11.17)
6	5 (2.48)	7 (3.45)	11 (5.34)	7 (3.40)
7	7 (3.47)	7 (3.45)	10 (4.85)	12 (5.83)
8	9 (4.46)	4 (1.97)	16 (7.77)	15 (7.28)
9	4 (1.98)	2 (0.99)	9 (4.37)	2 (0.97)
10	79 (39.11)	9 (4.43)	90 (43.69)	30 (14.56)

Supplementary Table S1b. Illness perception dimensions

(continued)

Rating scale	How much is solar radiation responsible for the illness n (%)	Sunscreen use BEFORE diagnosis n (%)	Sunscreen use AFTER diagnosis n (%)	Financial burden n (%)
1	8 (3.86)	107 (51.94)	16 (7.73)	121 (58.17)
2	0 (0)	16 (7.77)	6 (2.90)	32 (15.38)
3	2 (0.97)	13 (6.31)	6 (2.90)	9 (4.33)
4	2 (0.97)	6 (2.91)	4 (1.93)	5 (2.40)
5	8 (3.86)	16 (7.77)	15 (7.25)	20 (9.62)
6	8 (3.86)	5 (2.43)	1 (0.48)	3 (1.44)
7	11 (5.31)	5 (2.43)	6 (2.90)	2 (0.96)
8	24 (11.59)	8 (3.88)	27 (13.04)	5 (2.40)
9	15 (7.25)	2 (0.97)	11 (5.31)	0 (0)
10	129 (62.32)	28 (13.59)	115 (55.56)	11 (5.29)

Supplementary Table S2. Treatment perception dimensions

Rating scale	Perception of treatment plan n (%)	Ease to comply with treatment n (%)	Feeling of control by treatment n (%)
1	8 (3.88)	2 (0.97)	1 (0.49)
2	6 (2.91)	1 (0.49)	3 (1.46)
3	9 (4.37)	3 (1.46)	1 (0.49)
4	4 (1.94)	2 (0.97)	0 (0)
5	11 (5.34)	10 (4.85)	7 (3.40)
6	2 (0.97)	2 (0.97)	4 (1.94)
7	11 (5.34)	9 (4.37)	10 (4.85)
8	19 (9.22)	23 (11.17)	23 (11.17)
9	11 (5.34)	16 (7.77)	17 (8.25)
10	125 (60.68)	138 (66.99)	140 (67.96)

Supplementary Table S3. Treatment concerns according to gender

Concern about treatment	Males (n=168) n* (%)	Females (n=39) n* (%)
Duration	53 (31.55)	9 (23.08)
Safety	15 (8.93)	4 (10.26)
Efficacy	37 (22.02)	14 (35.9)
Pain/skin reaction	8 (4.76)	6 (15.38)
Cosmetic outcome	9 (5.36)	8 (20.51)
Cost	0 (0)	1 (2.56)
None of the above	62 (36.9)	11 (28.21)

**They do not add up to 208 patients &100%, because some patients gave more than one choice*

Supplementary Table S4. Prioritization of causal attributions

	n (%)				
**1st most important factor**	Solar radiation	Ageing	Stress	Nutrition/Alcohol/Smoking	Pollution
	163 (79.9)	11 (5.39)	9 (4.41)	8 (3.92)	6 (2.94)
**2nd most important factor**	Ageing	Stress	Pollution	Nutrition/Alcohol/Smoking	Solar radiation – Accidentally*
	64 (40)	34 (21.25)	23 (14.38)	11 (6.88)	10 (6.25)
**3rd most important factor**	Ageing	Pollution	Stress-Accidentally*	Solar radiation-sunscreens*	Vitamin D defeciency
	32 (24.06)	26 (19.55)	16 (12.03)	14 (10.53)	11 (8.27)

**Factors with the same percentage rates*

Supplementary Table S5. Causal attributions according to gender n (%)

**Causal attributions**	**Males**			**Females**		
	1st most important factor	2nd most important factor	3rd most important factor	1st most important factor	2nd most important factor	3rd most important factor
Solar radiation	133(81.1)	0 (0)	0 (0)	30 (75)	4 (12,9)	4 (13,33)
Stress	7 (4.27)	24 (18.6)	0 (0)	2 (5)	10(32.26)	4 (13.33)
Ageing	8 (4.88)	57(44.19)	19(18.45)	3 (7,5)	7 (22.58)	13(43.33)
Pollution	0 (0)	19(14.73)	21(20.39)	2 (5)	4 (12.9)	5 (16.67)
Nutrition/ Alcohol/ Smoking	7 (4.27)	0 (0)	0 (0)	0 (0)	0 (0)	0 (0)
Bacteria/Viruses	0 (0)	0 (0)	0 (0)	0 (0)	0 (0)	0 (0)
Sunscreen	0 (0)	0 (0)	13(12.62)	0 (0)	0 (0)	0 (0)
Vit. D deficiency	0 (0)	0 (0)	0 (0)	0 (0)	0 (0)	0 (0)
Accidentally	0 (0)	9 (6.98)	15(14.56)	0 (0)	0 (0)	0 (0)

Supplementary Table S6a. Causal attributions according to educational level n (%)

**Causal attributions**	**Primary school**			**High school**		
	1st most important factor	2nd most important factor	3rd most important factor	1st most important factor	2nd most important factor	3rd most important factor
Solar radiation	27 (81.8)	0 (0)	0 (0)	64 (75.3)	7 (11.5)	7 (11.5)
Stress	1 (3.0)	4 (15.4)	1 (5.3)	5 (5.9)	12 (19.7)	5 (1.4)
Ageing	0 (0)	10 (38.5)	5 (26.3)	8 (9.4)	21(34.4)	8 (16.7)
Pollution	1 (3.0)	6 (23.1)	6 (31.6)	4 (4.7)	10 (16.4)	10 (20.8)
Nutrition/ Alcohol/ Smoking	3 (9.1)	3 (11.5)	0 (0)	2 (2.4)	4 (6.6)	1 (2.1)
Bacteria/Viruses	0 (0)	1 (3.8)	0 (0)	0 (0)	1 (1.6)	0 (0)
Sunscreen	1 (3.0)	0 (0)	4 (21.1)	1 (1.2)	1 (1.6)	4 (8.3)
Vit. D deficiency	0 (0)	1 (3.8)	2 (10.5)	1 (1.2)	0 (0)	5 (10.4)
Accidentally	0 (0)	1 (3.8)	1 (5.3)	0 (0)	5 (8.2)	8 (16.7)

Supplementary Table S6b. Causal attributions according to educational level n (%) (continued)

**Causal attributions**	**University / Technical studies**			**MSc / PhD title**		
	1st most important factor	2nd most important factor	3rd most important factor	1st most important factor	2nd most important factor	3rd most important factor
Solar radiation	51 (79.7)	3 (5.6)	0 (0)	21 (95.5)	0 (0)	1 (5.6)
Stress	3 (4.7)	13 (24.1)	6 (12.5)	0 (0)	5 (26.3)	4 (22.2)
Ageing	3 (4.7)	25 (46.3)	11 (22.9)	0 (0)	8 (42.1)	8 (44.4)
Pollution	1 (1.6)	5 (9.3)	9 (18.8)	0 (0)	2 (10.5)	1 (5.6)
Nutrition/ Alcohol/ Smoking	2 (3.1)	4 (7.4)	0 (0)	1 (4.5)	0 (0)	0 (0)
Bacteria/Viruses	1 (1.6)	0 (0)	1 (2.1)	0 (0)	0 (0)	0 (0)
Sunscreen	1 (1.6)	3 (5.6)	4 (8.3)	0 (0)	0 (0)	2 (11.1)
Vit. D deficiency	0 (0)	0 (0)	4 (8.3)	0 (0)	1(5.3)	0 (0)
Accidentally	1 (1.6)	1 (1.9)	5 (10.4)	0 (0)	3 (15.8)	2 (11.1)

Supplementary Table S7. Causal attributions according to skin cancer history n (%)

**Causal attributions**	**With skin cancer history**			**Without skin cancer history**		
	1st most important factor	2nd most important factor	3rd most important factor	1st most important factor	2nd most important factor	3rd most important factor
Solar radiation	38 (90.5)	0 (0)	3 (11.5)	123 (76.9)	9 (7.1)	11 (10.3)
Stress	1 (2.4)	4 (12.1)	7 (26.9)	8 (5.0)	30 (23.6)	9 (8.4)
Ageing	1 (2.4)	16 (48.5)	4 (15.4)	10 (6.3)	48 (37.8)	28 (26.2)
Pollution	1 (2.4)	4 (12.1)	3 (11.5)	5 (3.1)	19 (15.0)	23 (21.5)
Nutrition/ Alcohol/ Smoking	0 (0)	3 (9.1)	1 (3.8)	8 (5.0)	8 (6.3)	2 (1.9)
Bacteria/Viruses	0 (0)	1 (3.0)	0 (0)	1 (0.6)	1 (0.8)	1 (0.9)
Sunscreen	1 (2.4)	2 (6.1)	3 (11.5)	2 (1.3)	2 (1.6)	11 (10.3)
Vit. D deficiency	0 (0)	0 (0)	4 (15.4)	1 (0.6)	2 (1.6)	7 (6.5)
Accidentally	0 (0)	2 (6.1)	1 (3.8)	1 (0.6)	8 (6.3)	15 (14.0)

Supplementary Tables S8. Statistically significant correlations

Table S8a. Statistically significant correlations of gender (a)

	n (%)	n (%)	n (%)
**How many AK do you know that you have?**	2-3	4+	I do not know
Males	32 (19.28)	79 (47.59)	55 (33.13)
Females	18 (46.15)	15 (38.46)	6 (15.38)
**How many AK treatments do you have already?**	0	1-3	4+
Males	21 (12.5)	64 (38.1)	83 (49.4)
Females	2 (5.13)	24 (61.54)	13 (33.33)

Table S8b. Statistically significant correlations of gender (b)

	Median (25th – 75th percentiles)	Range
**How much does your illness affect you?**		
Males	3 (1-7)	1-10
Females	5 (1-10)	1-10
**Sunscreen use BEFORE diagnosis**		
Males	1 (1-4)	1-10
Females	5 (2-10)	1-10
**Sunscreen use AFTER diagnosis**		
Males	10 (5-10)	1-10
Females	10 (9-10)	1-10
**Financial burden**		
Males	1 (1-2)	1-10
Females	2 (1-5)	1-10

Table S8c. Statistically significant correlations of working or activities outdoors history

**Number of treatments**	**0**	**1-3**	**4+**
History of working/activities outdoors	7 (7.22)	37 (38.14)	53 (54.64)
No history of working/activities outdoors	16 (14.68)	50 (45.87)	43 (39.45)
**Likely to receive treatment if AK precancerous**	**Yes**	**No**	
History of working/activities outdoors	89 (92.71)	7 (7.29)	
No history of working/activities outdoors	85 (80.19)	21 (19.81)	
**Sunscreen use BEFORE diagnosis**	**Median (25th – 75th percentiles)**	**Range**	
History of working/activities outdoors	1 (1-4.5)	1-10	
No history of working/activities outdoors	2 (1-7)	1-10	

Table S8d. Tendency to statistically significant correlations of solarium use history

	Median (25th – 75th percentiles)	Range
**Sunscreen use BEFORE diagnosis**		
History of solarium	6 (1-10)	1-10
No history of solarium	1 (1-5)	1-10
**Financial burden**		
History of solarium	2 (1-6)	1-10
No history of solarium	1 (1-2)	1-10
**Ease to comply with treatment**		
No history of solarium	10 (10-10)	6-10
No history of solarium	10 (8-10)	1-10
**Feeling of control of the disease by treatment**		
History of solarium	10 (10-10)	8-10
No history of solarium	10 (8.5-10)	1-10

Table S8e. Statistically significant correlations of skin cancer history

**Do you know the term AK?**	**Yes n (%)**	**No n (%)**
With skin cancer history	31 (72.09)	12 (27.91)
Without skin cancer history	86 (54.43)	72 (45.57)
**Likely to receive treatment, if about 0.5% of AK will turn into skin cancer**		
With skin cancer history	34 (85)	6 (15)
Without skin cancer history	97 (68.79)	44 (31.21)
	**Median (25th – 75th percentiles)**	**Range**
**Years from AK diagnosis**		
With skin cancer history	6 (2-10)	0-25
Without skin cancer history	2 (1-4)	0-26
**How long do you think your illness will last?**		
With skin cancer history	10 (5-10)	1-10
Without skin cancer history	5 (2-10)	1-10
**How well do you feel you understand your disease?**		
With skin cancer history	10 (6-10)	1-10
Without skin cancer history	8 (3-10)	1-10
**How much do you think solar radiation is responsible for your illness?**		
With skin cancer history	10 (8-10)	1-10
Without skin cancer history	10 (8-10)	1-10

Table S8f. Statistically significant correlations of level of education

Level of education	Do you know the term AK?		Understanding of the disease	Sunscreen use BEFORE diagnosis	Financial burden	Understanding of treatment plan	Ease to comply with treatment
	Yes n (%)	No n (%)	Median (Range)				
Primary school	14 (41.18)	20 (58.82)	4 (1-10)	1 (1-5)	2 (1-10)	8 (1-10)	9 (2-10)
High school	44 (53.66)	38 (46.34)	8 (1-10)	1 (1-10)	1 (1-10)	10 (1-10)	10 (1-10)
University / technical studies	44 (68.75)	20 (31.25)	9.5 (1-10)	3.5 (1-10)	1 (1-10)	10 (2-10)	10 (5-10)
MSc / PhD title	17 (73.91)	6 (26.09)	9.5 (1-10)	4 (1-10)	2 (1-6)	10 (1-10)	10 (7-10)

Table S8g. Statistically significant correlations of age

Age	Median (25th – 75th percentiles)	Range
Knowledge of term AK: Yes	69 (65-74)	34-85
Knowledge of term AK: No	72 (67-80)	40-88
Number of AK lesions: 2-3	68 (62-74)	34-86
Number of AK lesions: 4+	70 (66-75)	49-88
Number of AK lesions: I do not know	72.5 (67.5-78)	48-87

Table S8h. Spearman's Rho correlation (age)

	**Age**
**Understanding of the disease**	-0.124
**Sunscreen use BEFORE diagnosis**	-0.17
**Sunscreen use AFTER diagnosis**	-0.143
**Financial burden**	-0.127

Supplementary Table S9. Statistically significant correlations of knowledge of the term AK

	**Median (25th – 75th percentiles)**	**Range**	***p*-value**
**Understanding of the disease**			**<0.001**
Knowledge of the term AK: Yes	9 (6-10)	1-10	
Knowledge of the term AK: No	6 (1-10)	1-10	
**How long do you think your illness will last?**			**0.004**
Knowledge of the term AK: Yes	8 (3-10)	1-10	
Knowledge of the term AK: No	3 (1-10)	1-10	
**Understanding of treatment plan**			**0.044**
Knowledge of the term AK: Yes	10 (8-10)	1-10	
Knowledge of the term AK: No	10 (5-10)	1-10	

Supplementary Table S10. Statistically significant correlations of readiness for treatment, if positive in one of the two questions of information framing

	**Median (25th – 75th percentiles)**	**Range**	***p*-value**
**Concern about the disease**			**0.042**
Likely to receive treatment	3 (1-7)	1-10	
Not likely to receive treatment	1 (1-3)	1-10	
**Understanding of treatment plan**			**0.040**
Likely to receive treatment	10 (8-10)	1-10	
Not likely to receive treatment	8 (4-10)	2-10	
**Feeling of disease control by treatment**			*0.087*
Likely to receive treatment	10 (9-10)	1-10	
Not likely to receive treatment	10 (8-10)	3-10	

S1. Questionnaire

<u>**Demographics – Patient's characteristics**</u>

Choose the most representative answer for you or complete:

- Gender: Man / Woman

- Age:

- Education level:

1) Primary school completed
2) High school completed
3) University / technical studies
4) MSc / PhD title

- Do you work outdoors or have outdoor activities (now or in the past)? YES / NO

- Have you got skin cancer history? YES / NO

PART A: ILLNESS PERCEPTION QUESTIONS

A1. Choose the most representative answer for you or complete:

1. **Do you know the term actinic keratosis (AK)? YES / NO**
2. **Except from your dermatologist, you have heard/ are informed about AK from :**
    1) Doctor of another specialty
    2) Internet/television
    3) Pharmacy
    4) Friends / other contacts
    5) Family
    6) None of the above

3. **I was diagnosed with AK before… years**
4. **How many AK do you know that you have?**
    1) 2-3
    2) 4+
    3) I do not know
5. **How many times did you have already treatment for AK?**
    1) 0
    2) 1-3
    3) 4+
6. **Which of the following do you feel more familiar with?**
    1) Melanoma
    2) Basal cell Carcinoma
    3) Squamous cell Carcinoma
    4) Actinic Keratosis
    5) None of the above

A2. In the following questions please complete the number in a scale from 0 to 10, which best represents your opinion.

1. **How long do you think your illness will last?**
   1= very short time   10= forever

2. **How much do you experience symptoms from your illness (e.g., pain, itch)?**
   1= I feel absolutely nothing   10= very serious symptoms

3. **How well do you feel you understand your illness?**
   1= not at all   10= absolutely

4. **How much does your illness affect you (e.g., does it make you angry, scared, upset, depressed)?**
   1= not at all   10= extremely

5. **Please choose the three (3) most important factors, that according to your opinion are responsible for your illness and put them in prioritization order (putting numbers 1,2,3 aside):**

   1) Solar radiation
   2) Stress
   3) Ageing
   4) Pollution
   5) Nutrition/ Alcohol/ Smoking
   6) Bacteria/Viruses
   7) Sunscreen
   8) Vitamin D deficiency
   9) Accident
6. **How much do you think solar radiation is responsible for your illness?**
   1= not at all   10= absolutely

7. **How often did you use sunscreen BEFORE your AK diagnosis?**
   1= never   10= daily
8. **How often do you use / how willing are you to use sunscreen AFTER your AK diagnosis?**
   1= never   10= daily
9. **How much do you feel your illness affects (burdens) you financially?**
   1= not at all   10= extremely

PART B: INFORMATION FRAMING - AK TREATMENT

In the following questions please complete the number in a scale from 0 to 10 or choose the answer, which best represents your opinion.

1. **According to the following statement of your dermatologist, how likely would you be to follow treatment for AK? Answer with YES (likely to treat) or NO (not likely to treat)**
   A) AK are precancerous lesions.
   B) About 0.5% of AK turn into a non-life-threatening-skin cancer.

2. **How much do you feel you understand the suggested treatment plan for your illness?**
   1= not at all   10=absolutely
3. **How easy do you find to follow the suggested treatment plan?**
   1= impossible   10= absolutely easy
4. **How much do you think your treatment will help our illness?**
   1= not at all   10= absolutely
5. **What worries you the most about your illness?**
   1) Its duration
   2) Its safety
   3) Its efficacy
   4) Pain/ skin reaction
   5) Cosmetic Outcome
   6) Cost
   7) None of the above
